# The efficacy and safety of sodium nitroprusside in the treatment of schizophrenia: a meta-analysis

**DOI:** 10.3389/fpsyt.2023.1271624

**Published:** 2023-11-06

**Authors:** Xinxing Fei, Jiyang Li, Shiqi Wang, Jianxiong Wang, Changmei Guo, Rizhi Qisha, Yaqian Gao, Yue Hu

**Affiliations:** ^1^Department of Psychiatry, Chengdu Eighth People's Hospital (Geriatric Hospital of Chengdu Medical College), Chengdu, Sichuan, China; ^2^Department of Rehabilitation Medicine, Sichuan Tianfu New Area People's Hospital, Chengdu, Sichuan, China; ^3^Rehabilitation Medicine Center and Institute of Rehabilitation Medicine, West China Hospital, Sichuan University, Chengdu, China; ^4^Key Laboratory of Rehabilitation Medicine in Sichuan Province, Chengdu, Sichuan, China; ^5^Department of Rehabilitation Medicine, The Affiliated Hospital of Southwest Medical University, Luzhou, Sichuan, China; ^6^Department of Rehabilitation Medicine, The First Affiliated Hospital of Chengdu Medical College, Chengdu, Sichuan, China

**Keywords:** sodium nitroprusside, schizophrenia, meta-analysis, Positive and Negative Syndrome Scale, Brief Psychiatric Rating Scale

## Abstract

**Objective:**

Schizophrenia is a serious mental disease that brings not only serious burdens to patients and their families but also serious challenges to society. More research is needed to find better drugs to treat schizophrenia. This meta-analysis investigated the efficacy and safety of sodium nitroprusside in the treatment of schizophrenia.

**Methods:**

Randomized controlled trials comparing the efficacy and safety of sodium nitroprusside in the treatment of schizophrenia were searched via English and Chinese databases. The outcomes, including the Positive and Negative Syndrome Scale (PANSS) and Brief Psychiatric Rating Scale (BPRS), were recorded. RevMan 5.3 was used for the meta-analysis.

**Results:**

A total of six randomized controlled trials (174 patients) were included. The overall quality of the included studies was good. No statistically significant benefit of sodium nitroprusside over placebo was found when combined PANSS total and BPRS-18 (95% CI: −1.40, 0.02). Except for PANSS positive (95% CI: −1.86, −0.01), there was no significant difference in the scale score after sodium nitroprusside treatment compared with the control group in PANSS total (95% CI: −4.93, 0.23), PANSS general (95% CI: −2.53, 1.33), and PANSS negative (95% CI: −4.44, 0.89). The results of the sensitivity analysis excluding the study with clinical heterogeneity showed that sodium nitroprusside had no statistical benefit for the score of PANSS positive (95% CI: −2.19, 0.46). Moreover, there was also no significant difference in the BPRS-18 (95% CI: −3.23, −0.43).

**Conclusion:**

We conservatively believe that sodium nitroprusside does not alleviate the symptoms of schizophrenia compared with placebo. The subjects tolerated sodium nitroprusside well. Our findings provide a new idea for researchers to explore and solve the drug treatment of schizophrenia.

## Introduction

Schizophrenia is a serious mental disease with disorders in perception, thinking, emotion, and behavior that bring not only serious burdens to patients and their families but also serious challenges to society (1). The average lifetime morbid risk of schizophrenia was 11.9 per 1,000, with a slightly higher incidence in men (2). The incidence of the disease is higher in poorer and more dispersed social environments (3). Even worse, independent of familial factors, patients with mental disorders are at increased risk for subsequent complications (4). Thus, there is an urgent need to find effective treatments to improve the symptoms of schizophrenia.

Whether it is a positive or negative symptom of schizophrenia, there are different evaluations of the efficacy of nondrug therapy, and drug therapy is still the main treatment (5). To date, available antipsychotic drugs fail to meet clinical needs in the treatment of negative symptoms and cognitive impairment, and side effects caused by long-term use are gradually being reported (6, 7). Therefore, more research is needed to find better drugs to treat schizophrenia.

Sodium nitroprusside, a nitric oxide donor, is being investigated as a potential antipsychotic drug. The occurrence of schizophrenia is related to dysfunction of the N-methyl-D-aspartate receptor, and the expression level of nitric oxide is decreased (8, 9). In addition, cyclic guanosine monophosphate, one of the intermediate metabolites, is also a key molecule in the development of schizophrenia (9). Interestingly, sodium nitroprusside is an antihypertensive drug that releases blood vessels by releasing carbon monoxide, which might have the ability to regulate N-methyl-D-aspartate and intermediate metabolites (10).

Reports on the effectiveness of sodium nitroprusside have been inconsistent, including two previous randomized controlled trials published in *JAMA Psychiatry* (11, 12). Given that sodium nitroprusside does have the potential to treat schizophrenia, it is necessary to look for evidence in the treatment of schizophrenia with this drug.

## Methods

### Eligibility criteria

We used specific methods from our previously published protocol (13). In brief, randomized controlled trials of sodium nitroprusside in patients who were diagnosed with schizophrenia by the “Diagnostic and Statistical Manual of Mental Disorders” or “International Classification of Diseases” were included. Both Chinese and English trials were included, and there were no special requirements for the race, age, or gender of the subjects.

### Search strategy

The literature search followed the PRISMA reporting guidelines. We searched randomized controlled trials from English (PubMed, Web of Science, Embase, and Cochrane Library) and Chinese databases (China Biology Medicine disc, VIP, WanFang Data, and China National Knowledge Internet) using the keywords “sodium nitroprusside” and “schizophrenia.” The specific search strategy was listed in our previously published protocol (13). The final search time of all databases was May 21, 2023.

### Assessment of methodological quality and data synthesis

The same two investigators (XF and SW) independently extracted the data, and the third investigator (JL) participated in the discussion of conflicting data. The quality and risk of bias of the randomized controlled trials were evaluated according to the bias risk assessment tool of the *Cochrane Handbook for Systematic Reviews of Interventions*. The outcomes, including the Positive and Negative Syndrome Scale (PANSS) and Brief Psychiatric Rating Scale (BPRS), were synthesized by RevMan 5.3 (Cochrane Collaboration). The test level was set at two-sided *α = *0.05. Depending on the heterogeneity of the studies, we used either random- or fixed-effects models. Subgroup analysis and sensitivity analysis were performed.

## Results

### Literature search

**Figure 1:**
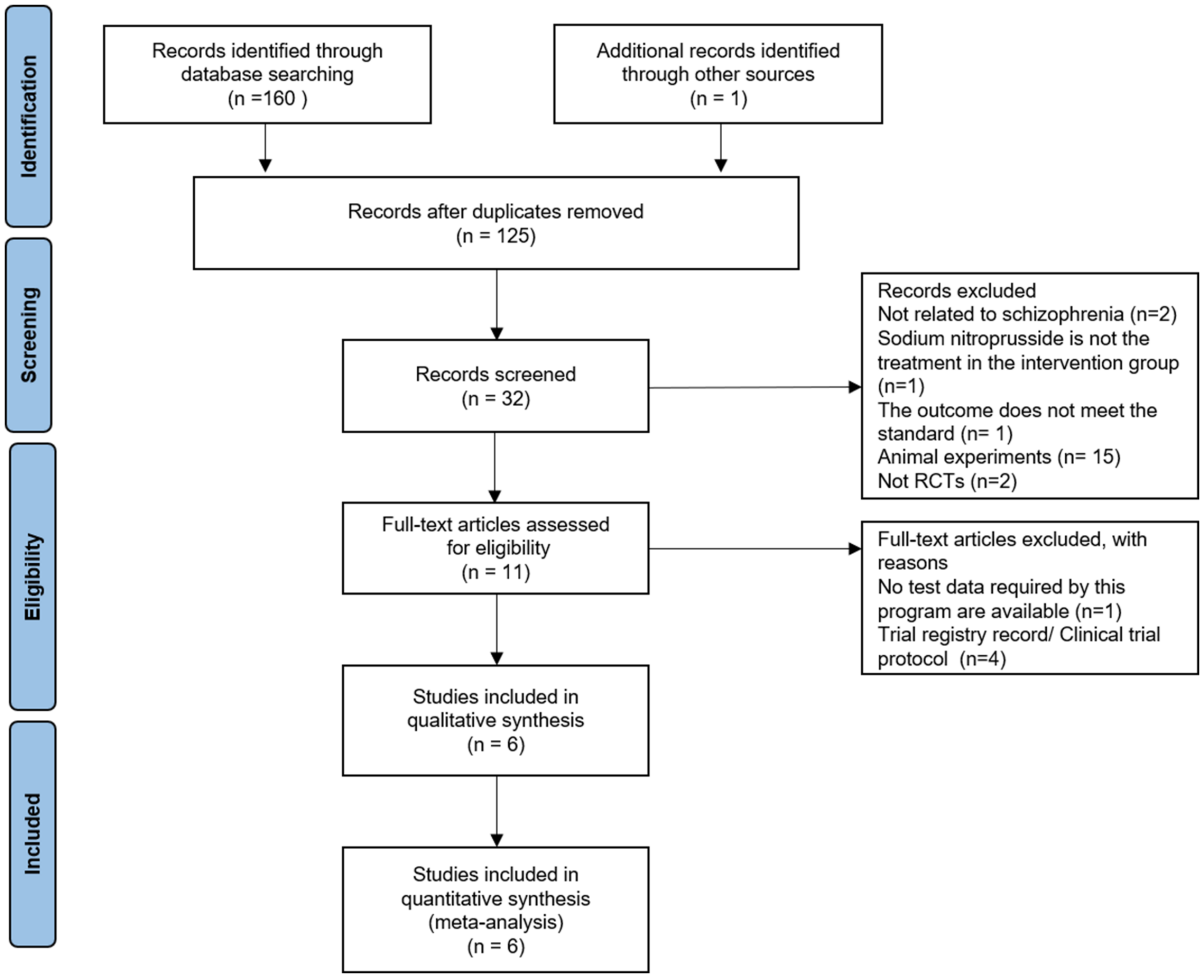
PRISMA flow diagram for the selection process.

A total of 161 articles were retrieved by searching the above databases. Finally, six randomized controlled trials (*n* = 174) (11, 12, 14–17) were included (Figure 1). Figure 1 shows the selection process for randomized controlled trials.

### The characteristics and quality of the included studies

**Figure 2:**
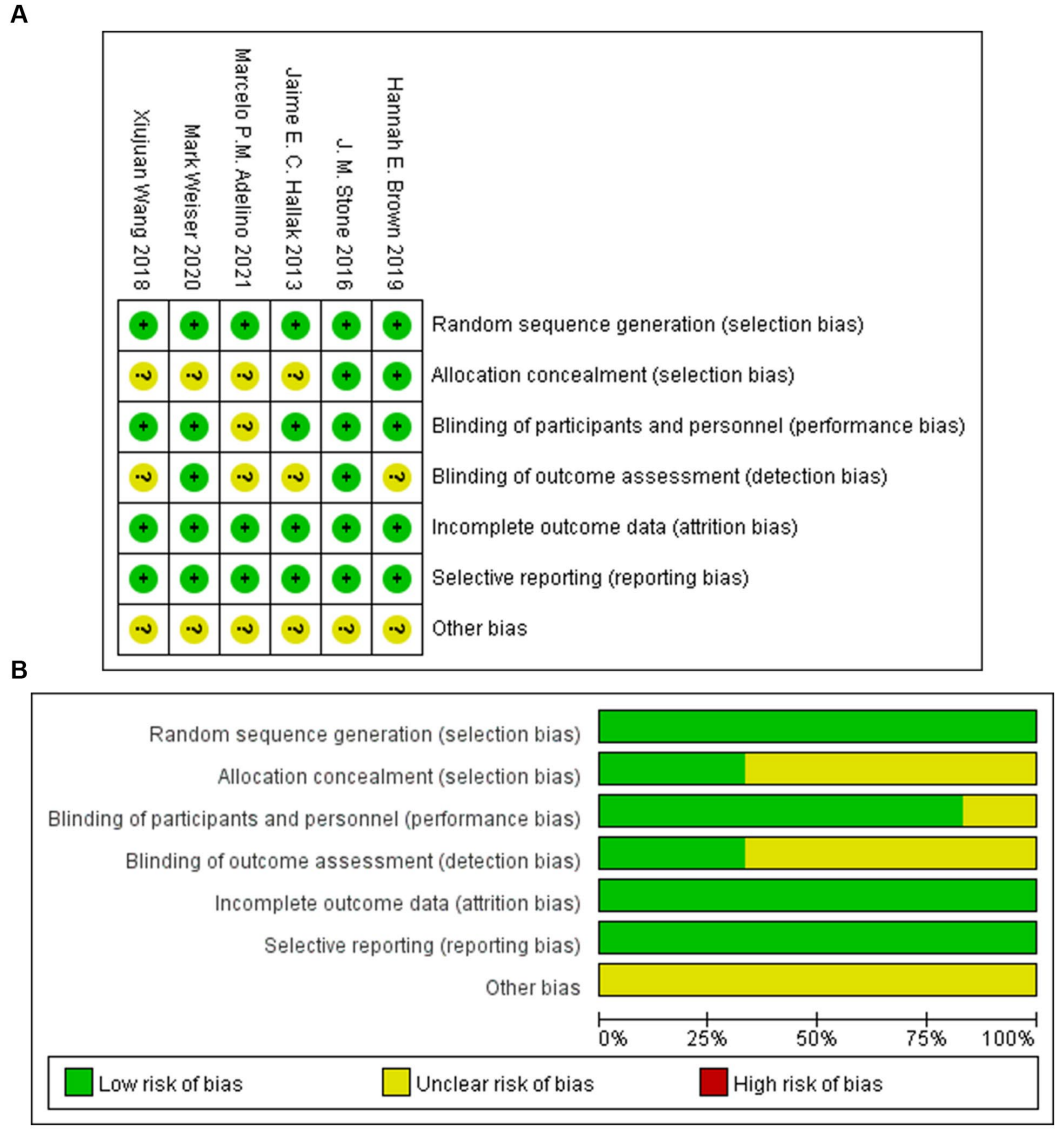
Summary **(A)** and graph **(B)** of the risk of bias.

The basic characteristics of the included studies are shown in Table 1. Six randomized controlled trials have compared the efficacy and safety of intravenous sodium nitroprusside with placebo for the treatment of schizophrenia. In all randomized controlled trials, the dosage of sodium nitroprusside was 0.5 μg/(kg·min). All studies selected the PANSS as the primary outcome, and three studies selected the BPRS-18 as a secondary outcome. The six included trials were all double-blind studies, and the overall quality of the included studies was good (Figure 2).

**Table 1:** Sodium nitroprusside for schizophrenia: Summary of studies included in this meta-analysis.

Study	Year	Country	Participants	Female sex	Age	Intravenous infusion [μg/(kg·min)]		Follow-up (weeks)	Outcomes
			Intervention/ Control			Sodium nitroprusside	Placebo		
Hallak et al.	2013	Canada	10/10	3/3	25.5 ± 6.7/25.6 ± 3.9	0.5	0.5	4	PANSS; BPRS-18
Stone et al.	2016	Britain	10/10	3/2	34 ± 9/40 ± 10	0.5	0.5	4	PANSS; BPRS-18
Wang et al.	2018	China	21/21	10/9	30.5 ± 7.3/29.4 ± 7.5	0.5	0.5	4	PANSS
Brown et al.	2019	United States	18/34	4/8	47.1 ± 10.5/43.0 ± 11.78	0.5	0.5	2	PANSS
Weiser et al.	2020	Moldova & Romania	10/10	3/7	36.1 ± 5.3/34.5 ± 3.6	0.5	0.5	4	PANSS
Adelino et al.	2021	Brazil	10/10	11^a^	43.4 ± 8.9^a^	0.5	0.5	4	PANSS; BPRS-18

a Gender and age in each group were not specified.

### PANSS total and BPRS-18

**Figure 3:**
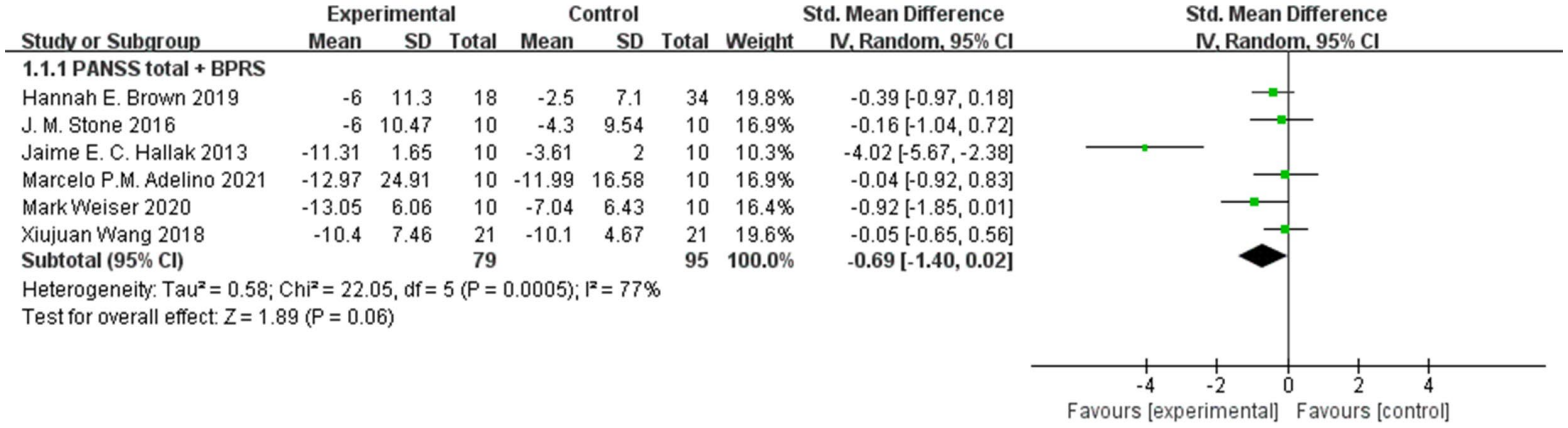
PANSS total and BPRS-18 scores in the comparison of sodium nitroprusside vs. placebo.

We combined the PANSS total and BPRS-18 scores in 6 trials, and we did not find that sodium nitroprusside had a statistically significant benefit over placebo (95% CI: −1.40, 0.02) (Figure 3). However, we also found that this synthesis led to high heterogeneity (I^2^ = 77%), so further subgroup analysis and sensitivity analysis were carried out.

#### PANSS

**Figure 4:**
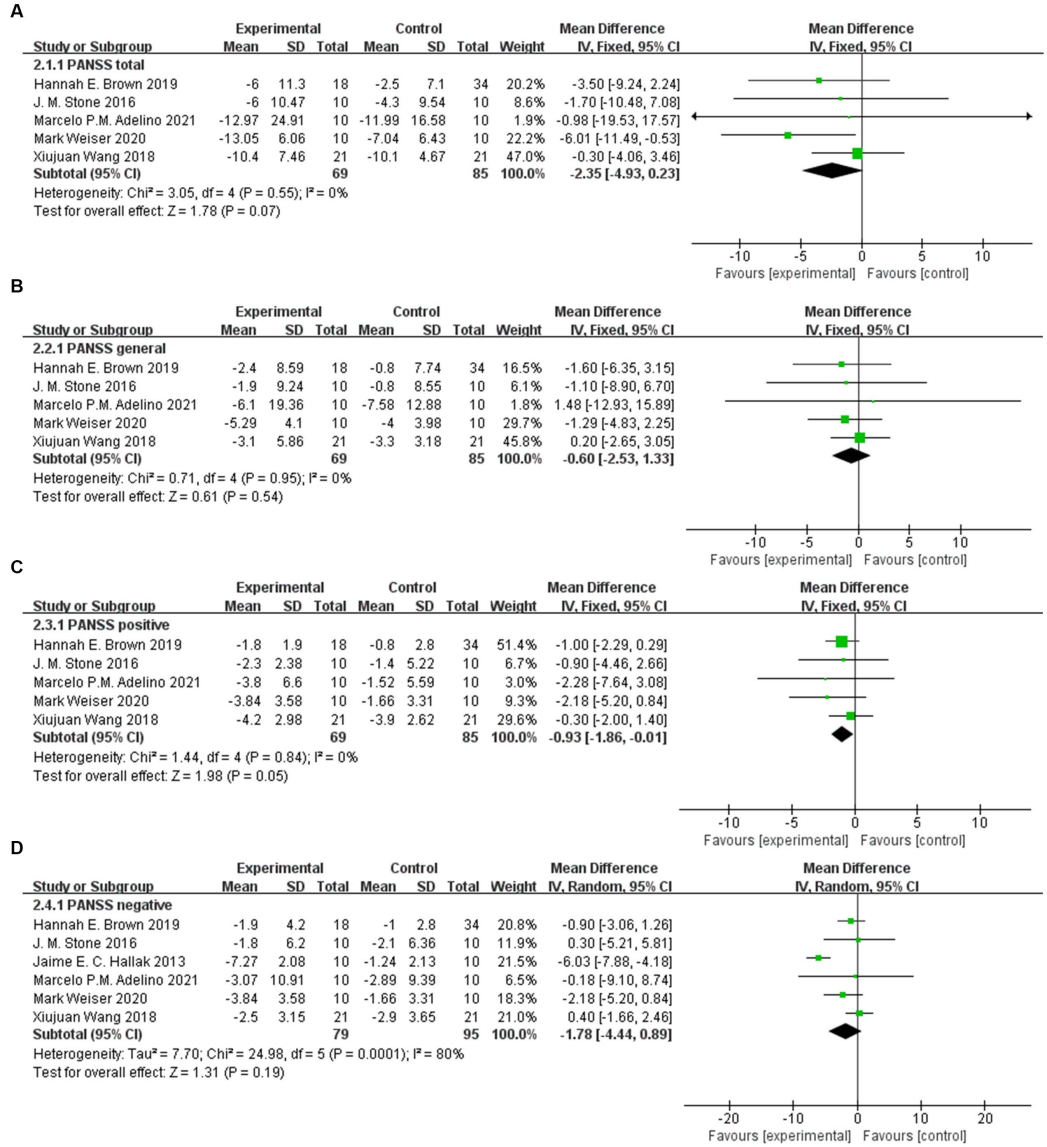
PANSS scores in the comparison of sodium nitroprusside vs. placebo. **(A)** PANSS total. **(B)** PANSS general. **(C)** PANSS positive. **(D)** PANSS negative.

Except for PANSS positive (95% CI: −1.86, −0.01), there was no significant difference in the scale score after sodium nitroprusside treatment compared with the control group in PANSS total (95% CI: −4.93, 0.23), PANSS general (95% CI: −2.53, 1.33), and PANSS negative (95% CI: −4.44, 0.89) (Figures 4A–D).

#### Sensitivity analysis of PANSS

**Figure 5:**
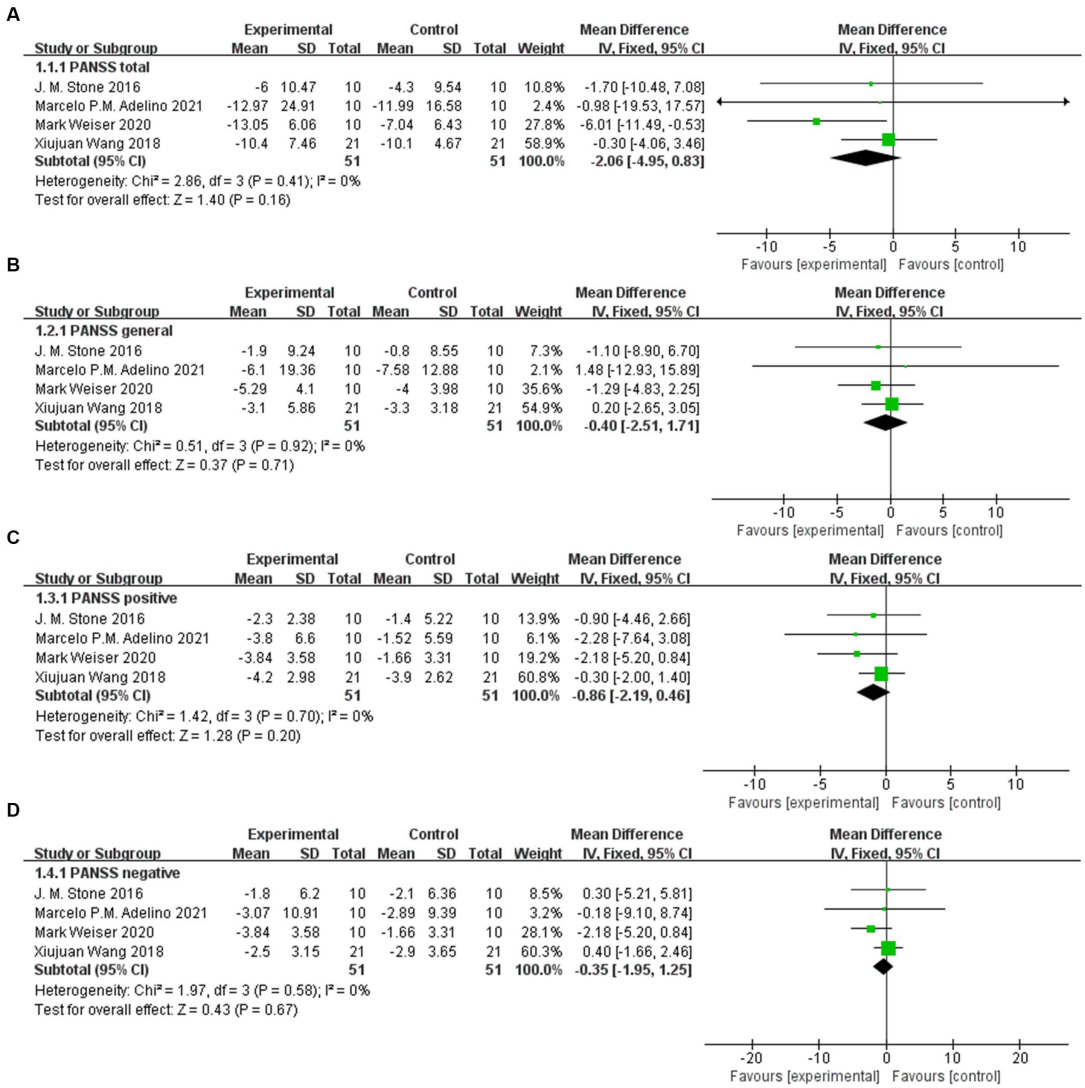
Sensitivity analysis of PANSS in the comparison of sodium nitroprusside vs. placebo. **(A)** PANSS total. **(B)** PANSS general. **(C)** PANSS positive. **(D)** PANSS negative.

The study of Hallak et al. (12) led to statistical heterogeneity after data consolidation, while the study of Brown et al. (11) had clinical heterogeneity (this randomized controlled trial assessed patients at 2 weeks). Therefore, we excluded these two studies from the additional analysis. The results of sensitivity analysis showed that there was still no significant difference in scale score between the two groups in PANSS total (95% CI: −4.95, 0.83), PANSS general (95% CI: −2.51, 1.71), PANSS positive (95% CI: −2.19, 0.46), and PANSS negative (95% CI: −1.95, 1.25) (Figures 5A–D).

#### BPRS-18

**Figure 6:**
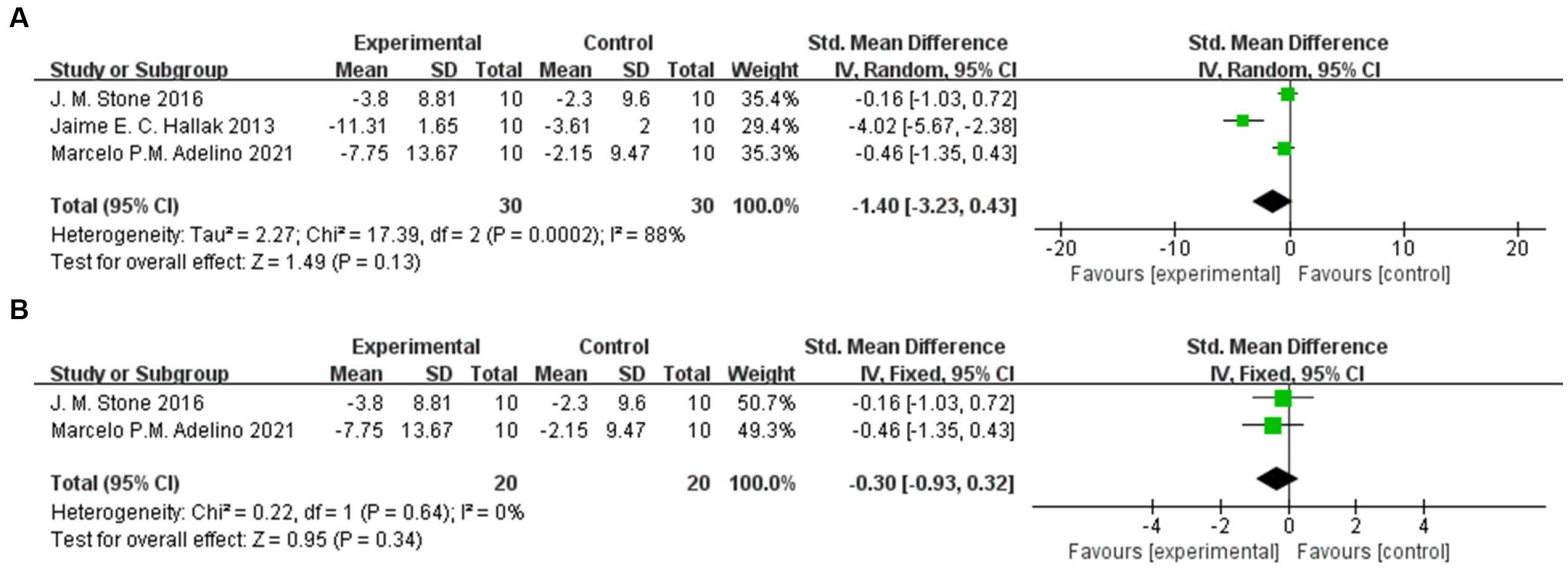
BPRS-18 scores in the comparison of sodium nitroprusside vs. placebo. **(A)** BPRS-18. **(B)** Sensitivity analysis of BPRS-18.

Three trials involving 60 patients were included, with 30 patients in the nitroprusside group and 30 patients in the placebo group. The combined results showed that there was no significant difference in BPRS-18 (95% CI: −3.23, −0.43) (Figure 6A). In the sensitivity analysis, we further eliminated one study that led to increased heterogeneity and finally included 2 studies for meta-analysis. Even so, there was no significant difference in BPRS-18 scores between the two groups (95% CI: −0.93, −0.32) (Figure 6B).

### Safety

In terms of safety, no serious adverse events were reported except for muscle spasms, asymptomatic hypotension, and venipuncture pain in some subjects. Most of the adverse events were relieved spontaneously during the intervention. Only one patient withdrew from the trial because of hypotension, and this patient’s data were not included in the final statistics (11).

## Discussion

Schizophrenia is an important public health problem, and finding drugs to treat its symptoms, especially negative symptoms, would be very beneficial to improve the quality of life of patients and reduce the burden on society. Schizophrenia is difficult to cure and requires long-term medication. The efficacy, safety, and tolerability of drugs are important factors in drug treatment. Sodium nitroprusside is a rapid, reliable, and easy-to-adjust antihypertensive drug that releases nitric oxide to directly relax arterioles and veins and is safe in clinical practice.

Sodium nitroprusside can eliminate the behavioral effect of phencyclidine and theoretically prevent the progression of mental symptoms (18). A previous review summarized the related mechanisms of sodium nitroprusside in the treatment of schizophrenia, such as the regulation of the NMDA-nNOS-cGMP signaling pathway, the relief of cerebral perfusion deficiency, the normalization of glutamate and dopaminergic neurotransmission function, and the strong antioxidant properties of the drug itself (19). Sodium nitroprusside is useful in treating drug-induced or spontaneous psychiatric symptoms in various preclinical studies. For example, ketamine-induced hyperkinesia was reduced after sodium nitroprusside intervention in rats (20). In addition, Diana et al. (21) found that early sodium nitroprusside treatment prevented behavioral abnormalities such as deficits in situational fear conditioning, hyperactivity, and social isolation in spontaneously hypertensive rats.

More importantly, some clinical studies have reported its efficacy in the treatment of schizophrenia, which is the main reason for our meta-analysis (22, 23). The original research by Hallak et al. (12) found that sodium nitroprusside was able to alleviate schizophrenia symptoms, but subsequent studies were unable to replicate this result. In our study, the initial synthetic results show that sodium nitroprusside is effective in the treatment of positive symptoms in patients with schizophrenia. This result is not consistent with the results of previous studies (17). More importantly, no study has reported that sodium nitroprusside can alleviate the positive symptoms of schizophrenia. Therefore, it is necessary to further analyze the characteristics of the included studies. We found that there was a clinical heterogeneity in the study published by Brown et al. (11) compared with other included studies, so we excluded this trial for additional analysis. Finally, we conservatively believe that both the primary outcome (PANSS) and secondary outcome (BPRS-18) did not change significantly after treatment with sodium nitroprusside.

At present, there are many scales for the evaluation of mental illness worldwide, but there are certain requirements for the selection of scales because of the complex symptoms of schizophrenia (24). The PANSS can evaluate the positive and negative symptoms and general conditions of patients with schizophrenia, which makes up for the shortcomings of poor sensitivity and accuracy of previous rating scales (25). The PANSS includes 7 items of the positive symptom scale, 7 items of the negative symptom scale, and 16 items of the General Psychopathology Scale. The scale assesses the presence and severity of psychiatric symptoms by the score of high or low, and the higher the score, the more serious the symptoms (26–28). Moreover, the BPRS-18 is widely used in clinical practice because of its few evaluation items and simple evaluation (29). Thus, to ensure the accuracy of outcome indicators and wide clinical applicability, the outcomes selected in our study were the PANSS, which has a more comprehensive evaluation range, and the BPRS-18 scale, which is commonly used in clinical practice. Of note, because the PANSS and BPRS-18 are highly correlated, we also combined the PANSS total and BPRS-18 findings before combining those for each scale separately.

Although we obtained a negative result in our study, this is more common in single-center studies than in multicenter trials. In the process of drug development, the probability of positive results in small single-center trials is higher than that in large-scale trials conducted in multiple centers (30). Slightly greater intervention effects have also been reported in single-center trials with continuous outcomes than in multicenter trials (31). The reasons for these results are manifold. First, single-center trials often have difficulty ensuring that enough samples are collected in a short period, which affects the follow-up statistical analysis to a certain extent. Second, some studies assume that the data are normally distributed. Due to the small sample size, it is difficult to ensure that the data completely conform to the normal distribution. Finally, the risk of bias is higher in single-center trials, which tend to include more homogeneous populations and may have more comprehensive research teams than multicenter trials (30, 32). These are important factors that cannot be repeated in the subsequent study, but we must admit that the initial study of Hallak et al. (12) has a great impact on the changes in psychopathology and promotes the development of the whole discipline.

There are some limitations in our study. Some studies did not introduce random and distribution hiding methods, which may have the possibility of bias. In addition, the included studies were only indexed journal articles that had been published and could be retrieved, which may have resulted in publication bias.

## Conclusion

In summary, our findings provide a new idea for researchers to explore and solve the drug treatment of schizophrenia. We conservatively believe that sodium nitroprusside does not alleviate the symptoms of schizophrenia compared with placebo. Additionally, the subjects tolerated sodium nitroprusside well. Necessarily, we hope that follow-up studies can provide more and higher quality clinical evidence.

## Data availability statement

The original contributions presented in the study are included in the article/supplementary material, further inquiries can be directed to the corresponding authors.

## Author contributions

XF: Data curation, Formal analysis, Methodology, Resources, Writing – original draft, Writing – review & editing. JL: Data curation, Formal analysis, Methodology, Writing – original draft, Writing – review & editing. SW: Data curation, Formal analysis, Methodology, Writing – original draft, Writing – review & editing. JW: Project administration, Resources, Writing – original draft, Writing – review & editing. CG: Writing – review & editing. RQ: Writing – review & editing. YG: Conceptualization, Project administration, Supervision, Writing – review & editing. YH: Conceptualization, Funding acquisition, Project administration, Supervision, Writing – review & editing.
